# Fibrosis is a common outcome following total knee arthroplasty

**DOI:** 10.1038/srep16469

**Published:** 2015-11-10

**Authors:** Nicole Abdul, David Dixon, Andrew Walker, Joanna Horabin, Nick Smith, David J. Weir, Nigel T. Brewster, David J. Deehan, Derek A. Mann, Lee A. Borthwick

**Affiliations:** 1 Fibrosis Research Group, Institute of Cellular Medicine, Newcastle University, Newcastle upon Tyne, NE2 4HH, UK; 2 Musculoskeletal Unit, Freeman Hospital, Newcastle Hospitals, NHS Trust, High Heaton, Newcastle upon Tyne, NE7 7DN, UK

## Abstract

Total knee arthroplasty (TKA) is one of the most successful orthopaedic procedures that alleviates pain and restores function in patients with degenerative knee joint diseases. Arthrofibrosis, abnormal scarring in which dense fibrous tissue prevents normal range of motion, develops in ~3–10% of TKA patients. No prophylactic intervention is available and treatment is restricted to aggressive physiotherapy or revision surgery. Tissue was collected from patients undergoing primary (n = 30) or revision (n = 27) TKA. Revision patients were stratified as non-arthrofibrotic and arthrofibrotic. Tissue was macroscopically and histologically compared to improve our understanding of the pathophysiology of arthrofibrosis. Macroscopically, tissue from primary TKA presents as homogenous, fatty tissue whereas tissue from revision TKA presents as dense, pigmented tissue. Histologically, there was dramatic tissue remodelling, increased collagen deposition and increased (myo)fibroblast staining in tissue from revision TKA. Significantly, tissue architecture was similar between revision patients regardless of clinically diagnosis. There are significant differences in architecture and composition of tissue from revision TKA over primary TKA. Surprisingly, whether revision TKA were clinically diagnosed as arthrofibrotic or non-arthrofibrotic there were still significant differences in fibrotic markers compared to primary TKA suggesting an ongoing fibrotic process in all revision knees.

Total knee arthroplasty (TKA) is one of the most successful orthopaedic procedures that reliably alleviates pain and restores function in patients who have degenerative knee joint diseases including osteoarthritis and rheumatoid arthritis. A recent audit revealed that 153,133 primary knee arthroplasties were performed in 396 centres throughout England and Wales in a 2-year period (01/07/2008–30/06/2010)^1^ and over 1.5 million are performed worldwide each year^2^. The surgery involves the resection of diseased or damaged bone from intra-articular areas of the knee followed by attachment of metal and polyethylene prosthetic replacements. Success rates for the operation are very high resulting in a substantial restoration of function and alleviation of pain and associated morbidity^3^. However, although a very high success rate is observed with TKA surgery, some 3–10% of individuals go on to develop fibrosis in the form of arthrofibrosis post-surgery^4^,^5^,^6^.

Arthrofibrosis, defined as abnormal scarring of the joint in which the formation of dense fibrous tissue and tissue metaplasia prevents normal range of motion, represents a significant clinical challenge and drastically reduces quality of life of those affected individuals^5^. Arthrofibrosis has been described as a fibrosing pathology of the synovial membrane^7^ and the infrapatellar fat pad^8^ with several studies highlighting an important role for the infrapatellar fat pad in regulating the function of the knee. For example Richmond and al Assal have described that Infrapatellar Contracture Syndrome (IPCS) is associated with knee surgery and that resection of the fibrotic fat pad can increase total range of movement^9^ and a recent review by Dragoo *et al.* highlights that inflammation and fibrosis within the infrapatellar fat pad caused by trauma and/or surgery, can lead to a variety of arthrofibrotic lesions including Hoffa’s disease, anterior interval scarring and IPCS^10^.

Fibroblasts are the most abundant cell in connective tissue and have been shown to play a critical role in physiological wound healing and restoration of tissue architecture. However, fibroblasts have also been shown to be one of the major effector cells involved in pathological wound repair and the development of fibrosis in multiple organs^11^,^12^,^13^,^14^. Significantly, the number of α-smooth muscle actin containing fibroblastic cells has been shown to be elevated in patients with arthrofibrosis after anterior cruciate ligament reconstruction suggesting a potentially important role for these cells in the development of fibrosis in the knee^15^.

To date, no prophylactic intervention is available and treatment for arthrofibrosis is restricted to aggressive physiotherapy or revision surgery^16^,^17^. Revision surgery puts the patient at risk of complications such as risks from undergoing an anaesthetic, infection, bleeding, damage to structures around the knee and pain amongst others. Revision surgery also comes at a great cost to the NHS including surgeon’s time, theatre use, theatre staff, equipment, postoperative high dependency bed and physiotherapy^18^.

Research investigating the mechanisms regulating/driving the development of arthrofibrosis following TKA is extremely limited and urgently needed to improve our understanding of the pathophysiology of arthrofibrosis and potentially lead to further studies investigating the feasibility of therapeutic targets to limit/reverse disease. In this study we macroscopically and histologically compare and contrast the tissue architecture and composition of the synovial membrane and infrapatellar fat pad of patients undergoing primary TKA, patients undergoing revision TKA for non-arthrofibrotic complications and patients undergoing revision TKA for the development of arthrofibrosis to improve our understanding of the pathophysiology of arthrofibrosis.

## Materials and Methods

### Patient recruitment and ethics

This study was performed in accordance with approval from the Newcastle and North Tyneside Local Regional Ethics Committee and informed written consent from all study patients (12/NE/0395).

### Tissue collection and processing

Infrapatellar fat pad and suprapatellar synovium (suprapatellar pouch) were collected from patients undergoing either primary TKA (n = 30) or patients undergoing revision TKA (n = 27). For the purposes of this study we chose these two surgical sites as reliable positions within the knee of early fibrotic response^7^,^8^,^9^,^10^. Patients undergoing revision TKA were stratified into non-arthrofibrotic revisions (n = 15) and arthrofibrotic revisions (n = 12). For the purposes of our study we accepted a definition of primary arthrofibrosis as occurring in the absence of infection (normal pre op CRP, apyrexial, no history of night sweats or of previous local or systemic infection), absence of component malalignment or gross over stuffing of the patellofemoral articulation, absence of heterotic ossification and/or evidence of shortening of the patella tendon on radio graphic assessment, patella tethering with thickened retinaculum, no neuromuscular disorder, no previous history of a fibrotic reaction in another joint or muscle complex, no history of dupuytren’s disease, no previous treatment for frozen shoulder, no features of reflex sympathetic dystrophy and with persistent loss of arc range of movement <90° when there had been a recorded history of greater active movement, and on table significant intra articular scarring present in 2 or more distinct anatomical sites^19^.

Tissue was stored at 4 °C in tissue culture media containing 10% FCS, 1% L-glutamine, 100units/ml penicillin and 100 μg/ml streptomycin prior to processing. Representative tissue biopsies (2× ~ 1 cm^3^) were fixed in 10% neutral buffered formalin overnight and paraffin embedded. Serial tissue sections (5 μm) were cut and processed for staining as described below.

### Picrosirius red staining

Tissue sections (5 μm) were deparaffinized in xylene, rehydrated in graded alcohol, and rinsed in water. Sections were treated in phosphomolybdic acid (0.2% aqueous) for 5 mins before staining in 0.1% sirius red F3B in saturated picric acid for 90 mins. After staining the sections were washed in 0.01N hydrochloric acid, dehydrated in graded alcohol and mounted in Pertex (3808706E Leica).

### Immunohistochemistry

Tissue sections (5 μm) were deparaffinized in xylene, rehydrated in graded alcohol, and incubated in 0.6% hydrogen peroxide/methanol for 15 minutes. Antigen retrieval was performed in proteinase K (20 μg/ml in PBS) for 30 mins at 37 °C or sodium citrate buffer (H3300, Vector Laboratories) for 20 mins in a microwave. Sections were blocked with avidin/biotin (SP-2001, Vector Laboratories) for 20 mins, then with 20% pig serum in PBS for 30 mins. Slides were incubated overnight at 4 °C with goat primary antibodies against collagen I (1/50 dilution - 1310-01, Southern Biotech), collagen III (1/50 dilution - 1330-01, Southern Biotech), vimentin (1/100 dilution, M7020, Dako) and fibronectin (1/250 dilution, F3648, Sigma) in 5% pig serum PBS or a FITC conjugated mouse monoclonal antibody against α-smooth muscle actin (α-SMA) (F3777, Sigma) at 1/1000 dilution in 5% pig serum PBS. Slides were washed with PBS and were incubated with biotinylated rabbit anti-sheep (BA-6000, Vector Laboratories) or biotinylated goat anti-fluorescein (BA-0601, Vector Laboratories) in 1% pig serum for 2 hours. Amplification of antigen was achieved using an R.T.U. Vectastain kit (PK-7100, Vector Laboratories) and positive staining was visualized by 3,3- diaminobenzidine tetrahydrochloride (SK-4100, Vector laboratories).

### Image analysis

Images were acquired on a Nikon Eclipse upright microscope. For image quantification, the mean percentage area positive for 10× randomly selected high powered fields (×10 magnification) was calculated using Nikon NIS elements image analysis software.

### Hydroxyproline assay

For assessment of fibrosis, in patients where sufficient tissue was available, hydroxyproline was measured as a surrogate for collagen content as previously described^20^. Briefly, 5–7 small biopsies (total weight ~200 mg) were collected from throughout the tissue and digested with 1ml of 6N HCl at 110 °C for 18 hours. Following digestion the sample is neutralised in 10N NaOH before colorization with Ehrlich’s reagent. A standard curve comprised of dilutions of 1mM hydroxyproline was used for quantification.

### Statistical analysis

Differences in the concentration of hydroxyproline (nmol/g) and the percentage (%) picrosirius red, collagen I, collagen III, vimentin, fibronectin and smooth muscle actin positive staining of synovial membrane and infrapatellar fat pad between patient subgroups (1. primary TKA patients & non-arthrofibrotic revision TKA patients, 2. primary TKA patients & arthrofibrotic revision TKA patients, and 3. non-arthrofibrotic revision TKA patients & arthrofibrotic revision TKA patients) were assessed in GraphPad prism version 6 (GraphPad Software, Dan Diego, CA) using a Mann-Whitney U-test. Differences with a p-value of  <0.05 were considered statistically significant.

## Results

### Patient selection and tissue acquisition

Patients undergoing primary TKA (n = 30) and revision TKA (n = 27) at Freeman hospital, Newcastle upon Tyne were consented to the study. Patients undergoing revision surgery were stratified as those without arthrofibrosis (n = 15) and those with arthrofibrosis (n = 12) (see Table 1).

**Figure 1:**
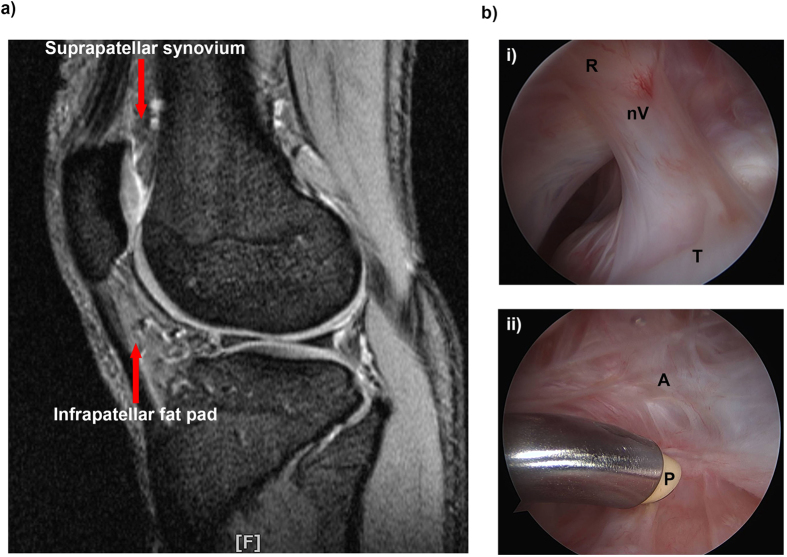
Arthroscopic evidence of arthrofibrosis in the knee. (**a**) Magnetic resonance image (MRI) of a knee joint. Red arrows indicate the location of the suprapatellar synovium and infrapatellar fat pad. (**b**) Arthroscopic view of an aberrant fibrous band bridging the space between the suprapatellar pouch roof (R) with evidence of neovascularisation (nV) and the superior articular margin of the trochlea (T) in a patient with severe arthrofibrosis (i). Surgical cautery with a diathermy probe (P) of dense layered adhesions (A) lying within the lateral synovial gutter of the knee (ii).

After eversion of the patella, prior to bone cuts (primary) or implant removal (revision) the infrapatellar fat pad or residuum is sectioned longitudinally whilst preserving the patellar tendon. This tissue is cautiously elevated off the tendon with a scalpel and removed from both the medial and lateral halves judiciously. In the native setting the suprapatellar pouch is identified centrally above the trochlea lying deep to the overlying quadriceps apparatus. In the revision setting it is visualised as an adherent anatomically equivalent adnexal mass lying above the primary femoral component anteriorly. See Fig. 1a for a visual representation of the location the tissues are sampled from.

### Macroscopic appearance of an arthrofibrotic knee

Figure 1bi represents an arthroscopic view of an aberrant fibrous band bridging the space between the suprapatellar pouch roof with evidence of neovascularisation in a patient with severe arthrofibrosis. Figure 1bii represents an arthroscopic view of surgical cautery with a diathermy probe of dense layered adhesions lying within the lateral synovial gutter of the knee.

**Figure 2:**
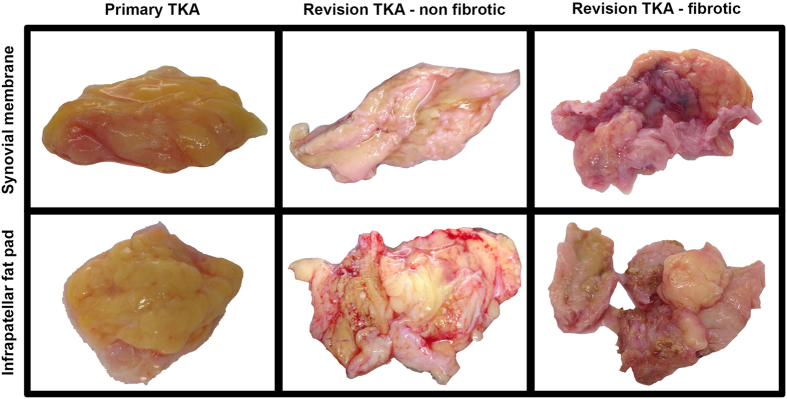
Macroscopic evaluation of tissue from primary and revision TKA. Synovial membrane (upper panel) and infrapatellar fat pad (lower panel) were isolated from patients undergoing primary TKA (left panel), patients undergoing non-arthrofibrotic revision TKA (middle panel) and patients undergoing arthrofibrotic revision TKA (right panel). Both tissues from primary TKA patients appeared to be homogenous, fatty, non-fibrotic tissue. In contrast, tissue from revision TKA patients appeared to be dense, pigmented, fibrotic tissue regardless of clinical diagnosis. Images acquired by Dr Lee Borthwick.

Macroscopically, the synovial membrane and infrapatellar fat pad collected from primary TKA patients appeared to be homogenous, fatty tissue (Fig. 2, left panel). In contrast, tissue from patients undergoing non-arthrofibrotic revision TKA (Fig. 2, middle panel) and patients undergoing arthrofibrotic revision TKA (Fig. 2, right panel) appeared to be dense, pigmented tissue. In additional primary tissue would float in tissue culture media whereas revision tissue was, for the majority of samples, submerged confirming an increased density in the revision tissue.

### Increased collagen deposition in revision TKA

**Figure 3:**
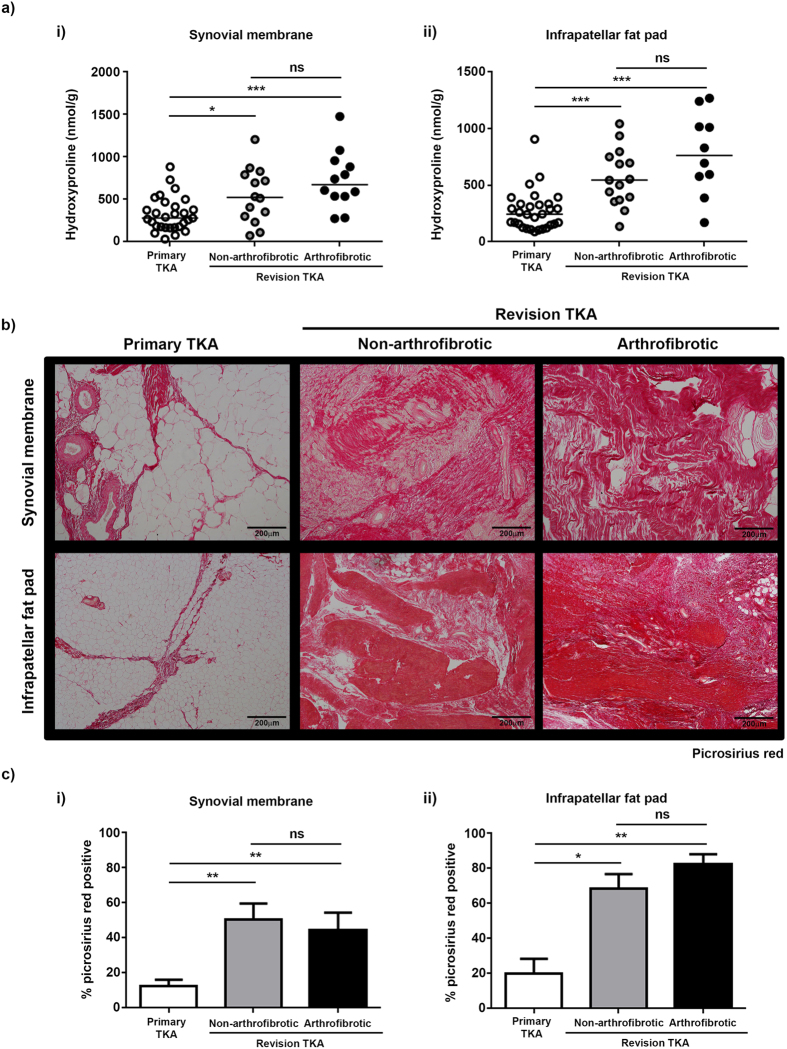
Significant tissue remodelling and collagen deposition following TKA. (a) In patients where sufficient tissue was available, hydroxyproline was measured as a surrogate for collagen content in tissue. There was a significant increase in the hydroxyproline content of synovial membrane (i) and infrapatellar fat pad (ii) from revision patients compared to primary TKA. There was no significant difference in the hydroxyproline content of either tissue between patients undergoing non-arthrofibrotic revision TKA and patients undergoing arthrofibrotic revision TKA. Synovial membrane; primary TKA n = 29, non-arthrofibrotic revision TKA n = 14, arthrofibrotic revision TKA n = 12. Infrapatellar fat pad; primary TKA n = 28, non-arthrofibrotic revision TKA n = 15, arthrofibrotic revision TKA n = 10. *p < 0.05. ***p < 0.001. (**b**) Representative images of picrosirius red stained synovial membrane (upper panel) and infrapatellar fat pad (lower panel) from patients undergoing primary TKA (left panel), patients undergoing non-arthrofibrotic revision TKA (middle panel) and patients undergoing arthrofibrotic revision TKA (right panel). There is evidence of significant tissue remodelling characterised by the loss of fat cells and the deposition of large quantities of densely packed collagen fibres in both tissues isolated from patients undergoing revision TKA regardless of clinical diagnosis. Images acquired on a Nikon inverted microscope at 10× magnification. (**c**) Picrosirius red positive staining was quantified using image analysis software. There was a significant increase in picrosirius red positive staining of synovial membrane (i) and infrapatellar fat pad (ii) from revision patients compared to primary TKA regardless of clinical diagnosis. There was no significant difference in picrosirius red positive staining of either tissue between patients undergoing non-arthrofibrotic revision TKA and patients undergoing arthrofibrotic revision TKA. Synovial membrane and Infrapatellar fat pad; primary TKA n = 9, non-arthrofibrotic revision TKA n = 10, arthrofibrotic revision TKA n = 10. *p < 0.05, **p < 0.01.

To quantify the degree of fibrosis in the tissue from primary and revision TKA patients we measured hydroxyproline content as a surrogate for levels of collagen. We found a significant increase in the tissue concentration of collagen in patients undergoing non-arthrofibrotic revision TKA (synovial membrane - 523 (74–1206) nmol/g, p = 0.03; infrapatellar fat pad - 546 (135–1042) nmol/g, p < 0.001) and patients undergoing arthrofibrotic revision TKA (synovial membrane - 674 (274–1478) nmol/g; p < 0.001; infrapatellar fat pad - 763 (170–1269) nmol/g, p < 0.001) compared to primary TKA (synovial membrane - 281 (32–883) nmol/g; infrapatellar fat pad - 244 (89–907) nmol/g). Surprisingly, there was no statistically significant difference (synovial membrane - p = 0.16; infrapatellar fat pad – p = 0.12) in the concentration of collagen between patients undergoing non-arthrofibrotic revision TKA and patients undergoing arthrofibrotic revision TKA, although a trend towards increased concentration of collagen in patients undergoing arthrofibrotic revision TKA was observed (Fig. 3a).

**Figure 4:**
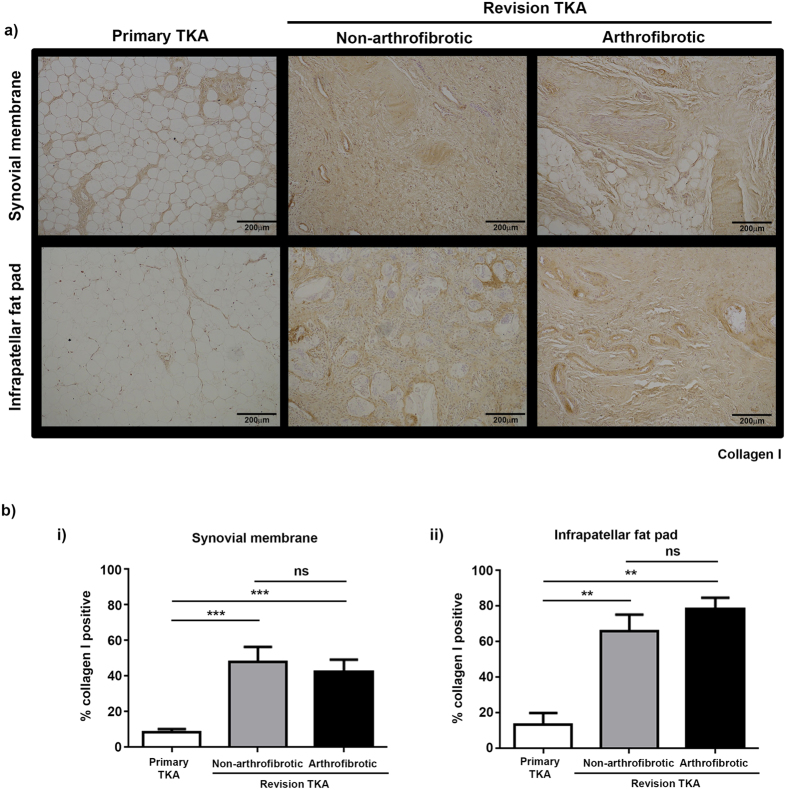
Collagen I is significantly increased in tissue from revision TKA. Representative images of collagen I stained synovial membrane (upper panel) and infrapatellar fat pad (lower panel) from patients undergoing primary TKA (left panel), patients undergoing non-arthrofibrotic revision TKA (middle panel) and patients undergoing arthrofibrotic revision TKA (right panel). There is a significant increase in collagen I expression in revision TKA tissue compared to primary TKA tissue. Images acquired on a Nikon inverted microscope at 10× magnification. (**b**) Collagen I positive staining was quantified using image analysis software. There was a significant increase in collagen I positive staining of synovial membrane (i) and infrapatellar fat pad (ii) from revision patients compared to primary TKA regardless of clinical diagnosis. There was no significant difference in collagen I positive staining of either tissue between patients undergoing non-arthrofibrotic revision TKA and patients undergoing arthrofibrotic revision TKA. Synovial membrane and Infrapatellar fat pad; primary TKA n = 9, non-arthrofibrotic revision TKA n = 10, arthrofibrotic revision TKA n = 10. **p < 0.01, ***p < 0.001.

**Figure 5:**
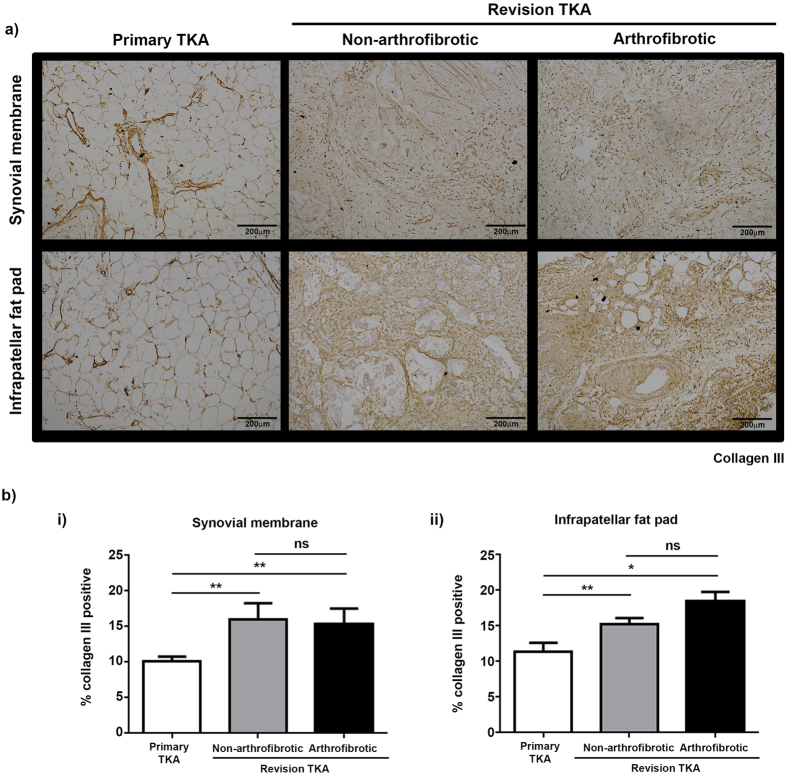
Increased collagen III expression in revision TKA tissue. Representative images of collagen III stained synovial membrane (upper panel) and infrapatellar fat pad (lower panel) from patients undergoing primary TKA (left panel), patients undergoing non-arthrofibrotic revision TKA (middle panel) and patients undergoing arthrofibrotic revision TKA (right panel). Collagen III expression is significant increase in revision TKA tissue compared to primary TKA tissue. Images acquired on a Nikon inverted microscope at 10× magnification. (**b**) Collagen III positive staining was quantified using image analysis software. There was a significant increase in collagen III positive staining of synovial membrane (i) and infrapatellar fat pad (ii) from revision patients compared to primary TKA regardless of clinical diagnosis. There was no significant difference in collagen III positive staining of either tissue between patients undergoing non-arthrofibrotic revision TKA and patients undergoing arthrofibrotic revision TKA. Synovial membrane and Infrapatellar fat pad; primary TKA n = 9, non-arthrofibrotic revision TKA n = 10, arthrofibrotic revision TKA n = 10. *p < 0.05, **p < 0.01.

Picrosirius red staining (Fig. 3b,c) and immunohistochemistry for collagen I (Fig. 4) and collagen III (Fig. 5) were used to confirm the increase in collagen in revision TKA patients and to investigate its distribution within the tissue. Both the synovial membrane and infrapatellar fat pad isolated from primary TKA are characterised by a homogenous distribution of white adipocytes (fat cells) with small amounts of collagen I distributed around adipocytes (Figs 3b and 4 left panel) and fibrotic bands of collagen III distributed throughout the tissue (Figs 3b and 5 left panel). For all stains there is evidence of significant tissue remodelling characterised by the loss of adipocytes and the deposition of large quantities of densely packed collagen I (Figs 3b and 4 middle and right panel) and collagen III (Figs 3b and 5 middle and right panel) fibres in both patients undergoing non-arthrofibrotic revision TKA and patients undergoing arthrofibrotic revision TKA.

There was a significant increase in the percentage positive staining for picrosirius red (Fig. 3c), collagen 1 (Fig. 4b) and collagen III (Fig. 5b) in patients undergoing non-arthrofibrotic revision TKA (picrosirius red: synovial membrane – 50 (20–79) %, p < 0.01; infrapatellar fat pad – 68 (51–88) %, p < 0.05) (collagen I: synovial membrane – 46 (23–70) %, p < 0.001; infrapatellar fat pad – 66 (43–88) %, p < 0.01;) (collagen III: synovial membrane – 16 (11–24) %, p < 0.01; infrapatellar fat pad – 15 (13–18) %, p < 0.01;) and patients undergoing arthrofibrotic revision TKA (picrosirius red: synovial membrane – 44 (20–73) %, p < 0.01; infrapatellar fat pad – 82 (68–95) %, p < 0.01) (collagen I: synovial membrane – 42 (22–65) %, p < 0.001; infrapatellar fat pad – 79 (64–98) %, p < 0.01;) (collagen III: synovial membrane – 15 (12–24) %, p < 0.01; infrapatellar fat pad – 18 (16–24) %, p < 0.05;) compared to primary TKA (picrosirius red: synovial membrane - 13 (2.0–30) %; infrapatellar fat pad – 20 (2.4–89) %) (collagen I: synovial membrane – 8.6 (2.9–21) %; infrapatellar fat pad – 14 (2.9–71) %) (collagen III: synovial membrane – 10 (8–14) %; infrapatellar fat pad – 11 (8–21) %). There was no statistically significant difference in picrosirius red (synovial membrane - p = 0.53; infrapatellar fat pad – p = 0.22), collagen I (synovial membrane - p = 0.67; infrapatellar fat pad – p = 0.41), and collagen III (synovial membrane - p = 0.41; infrapatellar fat pad – p = 0.06) staining between patients undergoing non-arthrofibrotic revision TKA and patients undergoing arthrofibrotic revision TKA.

The comparable level and distribution of collagen seen between the two groups of revision TKA patients confirms the biochemical data generated above (Fig. 3a).

### Increased α-smooth muscle actin expression in revision TKA

**Figure 6:**
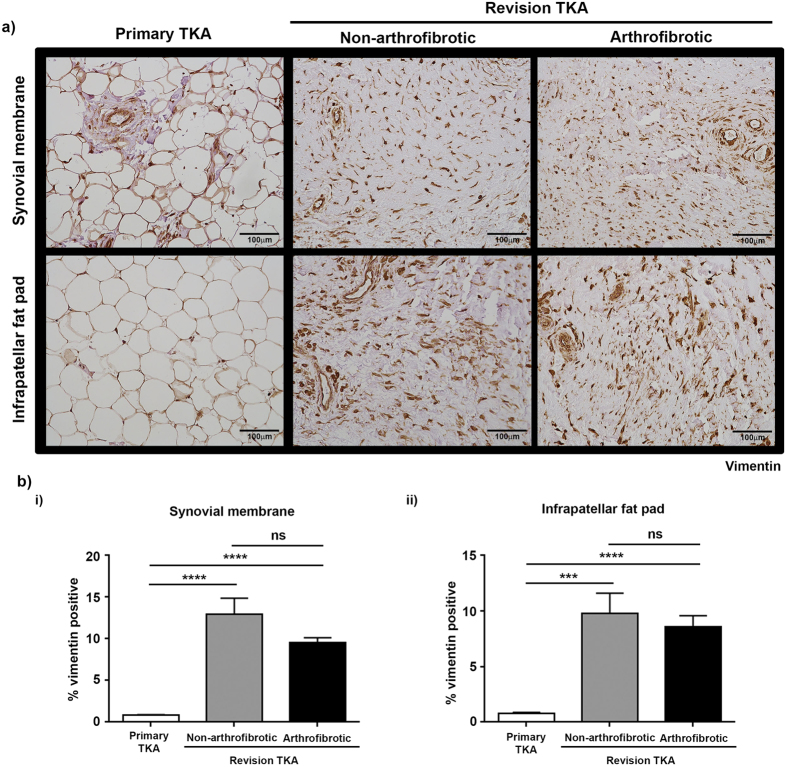
Increased expression of vimentin positive cells in tissue from revision TKA. (**a**) Representative images of vimentin stained synovial membrane (upper panel) and infrapatellar fat pad (lower panel) from patients undergoing primary TKA (left panel), patients undergoing non-arthrofibrotic revision TKA (middle panel) and patients undergoing arthrofibrotic revision TKA (right panel). Vimentin staining is increase in revision TKA tissue compared to primary TKA tissue. Images acquired on a Nikon inverted microscope at 20× magnification. (**b**) Vimentin positive staining was quantified using image analysis software. There was a significant increase in vimentin positive staining of synovial membrane (i) and infrapatellar fat pad (ii) from revision patients compared to primary TKA regardless of clinical diagnosis. There was no significant difference in vimentin positive staining of either tissue between patients undergoing non-arthrofibrotic revision TKA and patients undergoing arthrofibrotic revision TKA. Synovial membrane and Infrapatellar fat pad; primary TKA n = 9, non-arthrofibrotic revision TKA n = 10, arthrofibrotic revision TKA n = 10. ***p < 0.001, ****p < 0.0001.

**Figure 7:**
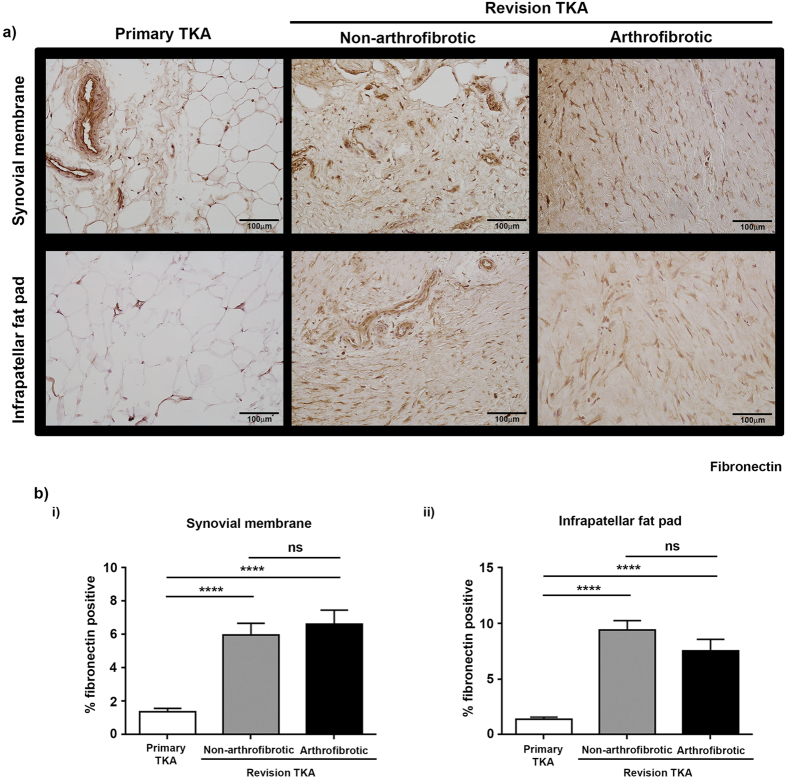
Increased expression of fibronectin positive cells in tissue from revision TKA. (**a**) Representative images of fibronectin stained synovial membrane (upper panel) and infrapatellar fat pad (lower panel) from patients undergoing primary TKA (left panel), patients undergoing non-arthrofibrotic revision TKA (middle panel) and patients undergoing arthrofibrotic revision TKA (right panel). Fibronectin staining is increase in revision TKA tissue compared to primary TKA tissue. Images acquired on a Nikon inverted microscope at 20× magnification. (**b**) Fibronectin positive staining was quantified using image analysis software. There was a significant increase in fibronectin positive staining of synovial membrane (i) and infrapatellar fat pad (ii) from revision patients compared to primary TKA regardless of clinical diagnosis. There was no significant difference in fibronectin positive staining of either tissue between patients undergoing non-arthrofibrotic revision TKA and patients undergoing arthrofibrotic revision TKA. Synovial membrane and Infrapatellar fat pad; primary TKA n = 9, non-arthrofibrotic revision TKA n = 10, arthrofibrotic revision TKA n = 10. ****p < 0.0001.

**Figure 8:**
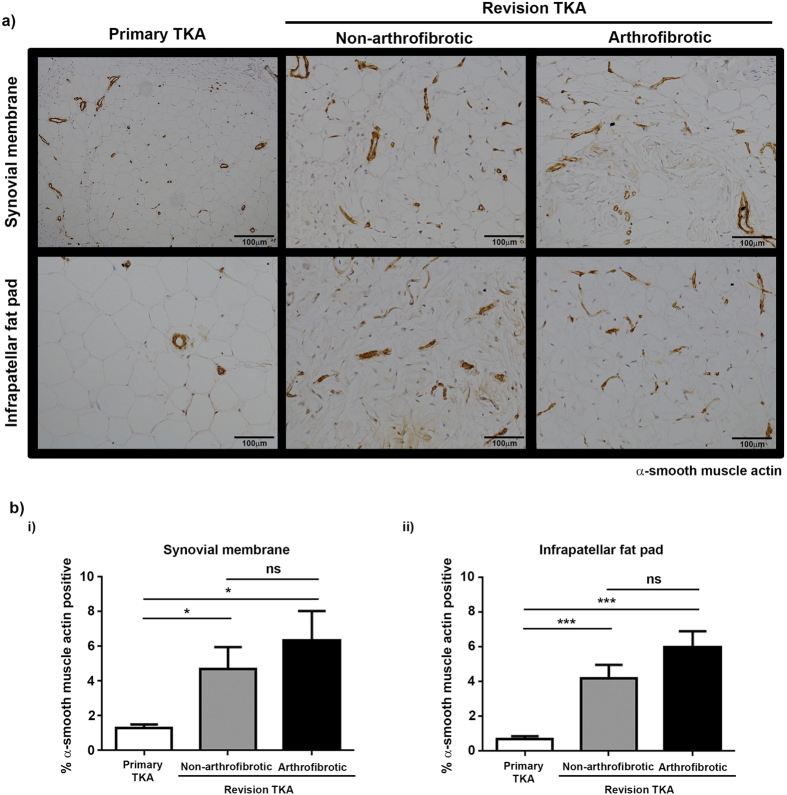
Increased expression of α-SMA positive cells in tissue from revision TKA. (**a**) Representative images of α-SMA stained synovial membrane (upper panel) and infrapatellar fat pad (lower panel) from patients undergoing primary TKA (left panel), patients undergoing non-arthrofibrotic revision TKA (middle panel) and patients undergoing arthrofibrotic revision TKA (right panel). α-SMA staining is increase in revision TKA tissue compared to primary TKA tissue. Images acquired on a Nikon inverted microscope at 20x magnification. (**b**) α-SMA positive staining was quantified using image analysis software. There was a significant increase in α-SMA positive staining of synovial membrane (i) and infrapatellar fat pad (ii) from revision patients compared to primary TKA regardless of clinical diagnosis. There was no significant difference in α-SMA positive staining of either tissue between patients undergoing non-arthrofibrotic revision TKA and patients undergoing arthrofibrotic revision TKA. Synovial membrane and Infrapatellar fat pad; primary TKA n = 10, non-arthrofibrotic revision TKA n = 5, arthrofibrotic revision TKA n = 5. *p < 0.05. ***p < 0.001.

Fibroblasts are cells of distinct morphology that are responsible for the maintenance and deposition of extracellular matrix throughout the body and play a critical role in physiological wound repair. However fibroblasts, in particular α-SMA positive myofibroblasts, have also been identified as one of the major effector cells in pathological wound repair and the subsequent development of fibrosis in multiple tissues/organs^12^,^13^,^14^. We therefore proceeded to stain synovial membrane and infrapatellar fat pad from primary and revision TKA for vimentin and fibronection to determine the level of fibroblasts present and α-SMA to determine the levels of myofibroblasts present. There was a significant increase in the percentage positive staining for vimentin (Fig. 6) and fibronectin (Fig. 7) in patients undergoing non-arthrofibrotic revision TKA (vimentin: synovial membrane – 13 (4.0–22) %, p < 0.0001; infrapatellar fat pad – 9.8 (0.6–19) %, p < 0.001) (fibronectin: synovial membrane – 6.0 (2.0–7.8) %, p < 0.0001; infrapatellar fat pad – 9.4 (6.8–15) %, p < 0.0001;) and patients undergoing arthrofibrotic revision TKA (vimentin: synovial membrane – 9.6 (7.1–12) %, p < 0.0001; infrapatellar fat pad – 8.6 (5.1–15) %, p < 0.0001) (fibronectin: synovial membrane – 6.6 (3.6–12.7) %, p < 0.0001; infrapatellar fat pad – 7.5 (2.9–12) %, p < 0.0001;) compared to primary TKA (vimentin: synovial membrane - 0.8 (0.4–1.3) %; infrapatellar fat pad – 0.8 (0.3–1.1) %) (fibronectin: synovial membrane – 1.4 (0–2.2) %; infrapatellar fat pad – 1.4 (0.1–2.3) %). Figure 8 shows that there was a significant increase in α-SMA positive staining in patients undergoing non-arthrofibrotic revision TKA (synovial membrane - 4.3 (0.8–8.7) %, p < 0.03; infrapatellar fat pad - 4.2 (2.5–6.7) %, p < 0.001) and patients undergoing arthrofibrotic revision TKA (synovial membrane - 8.1 (0.8–9.8) %, p < 0.03; infrapatellar fat pad - 6.2 (3.0–8.5) %, p < 0.001) compared to primary TKA (synovial membrane - 0.9 (0.6–2.4) %; infrapatellar fat pad – 0.5 (0.1–1.9) %, p < 0.001).

There was no statistically significant difference in vimentin (synovial membrane - p = 0.16; infrapatellar fat pad – p = 0.57), fibronectin (synovial membrane - p = 0.99; infrapatellar fat pad – p = 0.35), and α-SMA (synovial membrane - p = 0.67; infrapatellar fat pad – p = 0.15) staining between patients undergoing non-arthrofibrotic revision TKA and patients undergoing arthrofibrotic revision TKA.

## Discussion

Our data demonstrates that tissue isolated from patients undergoing revision TKA, regardless of clinical diagnosis, is macroscopically and histologically different from tissue isolated from patients undergoing primary TKA, with evidence of significant tissue remodelling characterised by loss/replacement of adipocytes with dense areas of collagen. Furthermore, regardless of whether revision patients were clinically stratified as non-arthrofibrotic or arthrofibrotic there was still dramatic evidence of tissue remodelling suggesting that an ongoing fibrotic process is occurring in most, if not all, revision knees. Validation of our observations in future studies with a larger patient cohort will be of great importance.

Significantly, and of great interest, is the question of why this fibrosis leads to a reduction in arc of motion in some patients and not others. To begin to address this question we must consider the limitations of this study. Firstly, our study has investigated the concentration of collagen in the tissue samples we received from the patients. This provides very accurate information regarding the density of collagen in the tissue but provides little detail regarding the total amount of collagen/scar tissue found in the knee. Perhaps the amount rather than the density of the fibrosis defines an effect on arc of motion? Furthermore we have so far only measured collagen content in two tissues and investigating fibrosis in other anatomical locations within the knee, for example the posterior capsule, is required. Addressing these questions in future studies will improve our understanding of the development of arthrofibrosis following TKA.

The infrapatellar fat pad occupies the space formed by the patella, patellar tendon, tibial plateau, and the femoral condyles^21^ and is only metabolised in severe malnutrition, implying an essential role in joint function^22^. In addition the infrapatellar fat pad had been hypothesised to play a key role in the supply of blood to the patellar tendon, the biomechanics of the knee joint, as well as being a modulator of inflammatory responses around the knee^23^,^24^,^25^. However, despite the evidence for an important biological role, the removal of the infrapatellar fat pad during TKA remains a matter of surgeon preference. A recent study from Moverley *et al.* investigated the 1 year postoperative outcomes of patients with total or partial excision of the infrapatellar fat pad versus patients in which the infrapatellar fat pad was preserved^26^. The data demonstrates that patients in which the infrapatellar fat pad was preserved had significantly better Oxford Knee Scores (OKS) associated with rising from a chair, pain, limping and giving way, further highlighting an important role for the infrapatellar fat pad *in vivo*. The infrapatellar fat pad was partially removed from all revision patients included in this study during their primary TKA. Our data suggests that the void in the intraarticular space created by the removal of the infrapatellar fat pad is being filled by either the pathological expansion/proliferation of the residual tissue and/or by the deposition of large amounts of extracellular matrix proteins such as collagen I/III. Research comparing the architecture of tissue from revision patients in which the infrapatellar fat pad was excised or preserved at primary TKA is required to further investigate this hypothesis. However it is clear that the infrapatellar fat pad is not simply an inert structure that can be ignored when we consider the development of arthrofibrosis.

Our data also highlights a significant increase in α-SMA positive staining in tissue isolated from revision TKA compared to primary TKA, indicating an increase in the number of myo-fibroblasts in the tissue. Our data is in agreement with Unterhauser *et al.* who reported a 10 fold increase in the frequency of α-SMA containing contractile fibroblastic cells in knee arthrofibrotic tissue after anterior crucial ligament reconstruction^15^. Furthermore there is a non-statistically significant trend towards an increase in α-SMA positive staining in tissue isolated from arthrofibrotic TKA compared to tissue isolated from non-arthrofibrotic TKA suggesting an ongoing fibrotic process and a potentially important role for these cells in disease pathology. Further investigating differences in protein and gene expression of key fibrogenic markers in tissue from primary and revision TKA will greatly improve our understanding of the remodelling process in the knee.

Currently no prophylactic intervention is available for arthrofibrosis and therefore treatment is restricted to aggressive physiotherapy or revision surgery. However an encouraging pilot study from Brown *et al.* demonstrated that intra-articular injection of Anakinra, an IL-1R antagonist, into patients with arthrofibrosis improved range of movement and swelling, with the majority also reporting a reduction in pain and a return to prior activity levels, however the mechanism of action is unknown^27^. Previous work in our laboratory has identified that fibroblasts isolated from human lung tissue express a high level of IL-1R and are strongly activated by treatment with IL-1α. Furthermore mice lacking IL-1α or IL-1R are protected from bleomycin induced fibrosis in the lung^28^. These observations, together with our data showing an increase in α-SMA positive cells in revision TKA, identifies a potential mechanism of action for Anakinra in arthrofibrosis that requires further investigation.

In summary there is a significant difference in the architecture and composition of the synovial membrane and infrapatellar fat pad of patients undergoing revision TKA compared to patients undergoing primary TKA. Surprisingly, whether revision TKA patients were clinically diagnosed as arthrofibrotic or non-arthrofibrotic there were still significant differences in fibrotic markers compared to primary TKA suggesting an ongoing fibrotic process is occurring in all revision knees. Further research is required to further understand the mechanisms driving tissue remodeling following TKA and to investigate why the presence of fibrosis in the knee leads to restricted range of motion in only a limited number of patients.

## Additional Information

**How to cite this article**: Abdul, N. *et al.* Fibrosis is a common outcome following total knee arthroplasty. *Sci. Rep.* **5**, 16469; doi: 10.1038/srep16469 (2015).

## Figures and Tables

**Table 1:** Patient demographics.

	Sex (M:F)	Age (median(range))	Time from primary to revision TKA (median(range))	Primary indication for surgery
Primary TKA (n = 30)	17M:13F	66 years (44–81)	N/A	Osteoarthrosis (n = 29), Rheumatoid disease (n = 1)
Non-arthrofibrotic revision TKA (n = 15)	5M:10F	74 years (40–82)	10 years (2–20)	Osteolysis and loose components (n = 9), Primary laxity pattern with functional instability (n = 3), Progression of osteoarthrosis (previous patellofemoral resurfacing) (n = 2), Revision for femoral medial condyle nonunion and pain (n = 1)
Arthrofibrotic revision TKA (n = 12)	6M:6F	69 years (44–88)	4 years (3–10)	Loss of movement with functional deficit, significant pain with activity (n = 11), Loss of movement with functional deficit, significant pain with activity and significant bone loss (n = 1)
